# Supplementary material for: Elucidating microbial iron corrosion mechanisms with a hydrogenase‐deficient strain of Desulfovibrio vulgaris
Source: mLife. 2024 Jun 28;3(2):269–76. doi: 10.1002/mlf2.12133 (PMC11211667; doi:10.1002/mlf2.12133)
Supplement: Supplementary file 1 — Supporting information. [file MLF2-3-269-s001.docx]

**Supplementary Material**

**Elucidating Microbial Iron Corrosion Mechanisms with a Hydrogenase-Deficient Strain of *Desulfovibrio vulgaris***

Di Wang^1,2^, Toshiyuki Ueki^1,2^, Peiyu Ma^1,2^, Dake Xu^1,2,*^ & Derek R. Lovley^1,3,*^

^1^Electrobiomaterials Institute, Key Laboratory for Anisotropy and Texture of Materials (Ministry of Education), Northeastern University, Shenyang, China

^2^Shenyang National Laboratory for Materials Science, Northeastern University, Shenyang, China

^3^Department of Microbiology, University of Massachusetts, Amherst, MA, USA

*Correspondence: [xudake@mail.neu.edu.cn](mailto:xudake@mail.neu.edu.cn), dlovley@umass.edu

Calculation of the H_2_ Concentration Required For Iron Oxidation Coupled to H_2_ Production to be Thermodynamically Unfavorable

In anaerobic anodic corrosion Fe^0^ is oxidized to Fe^2+^:

Fe^0^ → Fe^2+^ + 2 e^−^ (Reaction 1) *E*^0^ = −0.447 V (1)

where *E*^0^ is the standard potential.

The proton reduction reaction is:

H_2_ → 2 H^+^ + 2 e^−^ (Reaction 2) *E*^0^ = 0 V (2)

where *E*^0^ is the standard hydrogen electrode at 25 ^o^C, 1.0 M H^+^ and a H_2_ gas pressure of one atmosphere.

The reaction for the oxidation of Fe^0^ coupled to H_2_ production is:

Fe^0^ + 2 H^+^ → Fe^2+^ + H_2_ (Reaction 3) (3)

Thus, the potential for Reaction 3 is:

*E*^0^_reaction#3_ = *E*^0^_reduction_ − *E*^0^_oxidation_ = 0 V − (−0.447 V) = 0.447 V (4)

*E*_reaction#3_=*E*^0^_reaction#3_ − $\frac{RT}{nF}lnQ$ (5)

where *R* is the gas constant 8.314 J·K^−1^·mol^−1^, T is the temperature of 30 ^o^C for these studies, *n* is the number of moles of electrons exchanged (2), *F* is Faraday’s constant, 96485 J·V^−1^·mol^−1^, and Q is the reaction quotient of products and reactants.

Δ*G* = −*nFE*_reaction#3_ (6)

*E*_reaction#3_ = *E*^0^_reaction#3_  − $\frac{RT}{nF}lnQ$ = *E*^0^_reaction#3_ − $\frac{RT}{nF}ln\frac{p_{H2} c_{Fe2+}}{{(c_{H+})}^{2}}$ (7)

The maximum dissolved Fe^2+^ concentration possible in our incubations would have been generated with the parental strain, which exhibited the most corrosion. The total molar loss of Fe^0^ corroded by parental strain was 0.107 mmol in 30 mL of medium. Most of this the Fe^2+^ generated can be expected to precipitate as iron sulfide, which was abundant in the cultures. However, to be conservative, all the loss of Fe^0^ was considered to be oxidized to dissolved Fe^2+^. Thus, the actual inhibitory H_2_ concentration is expected to be higher than the number calculated here. In the 30 mL of medium, the ideal highest dissolved Fe^2+^ concentration would be 0.107 mmol/0.03mL = 3.57 mM = 0.00357 M. At pH 7 the concentration of H^+^ is 10^−7^ M.

*E*_reaction#3_ =$0.447- \frac{8.314 \times303.15}{2 \times96485}ln\frac{p_{H2} \times0.00357 M}{{({10}^{-7})}^{2} M}$

At equilibrium, Δ*G* = 0, and Δ*G* = −*nFE*_reaction#3_, so *E*_reaction#3_ = 0. Thus,

$$0=0.447 -\frac{8.314 \times303.15}{2 \times96485}ln\frac{p_{H2} \times0.00357 M}{{({10}^{-7})}^{2} M}$$

Rearranging:

$$\frac{8.314 \times303.15}{2 \times96485}ln\frac{p_{H2} \times0.00357}{{({10}^{-7})}^{2}}= 0.447$$

$ln\frac{p_{H2} \times0.00357}{{({10}^{-7})}^{2}}= \frac{0.447 \times2 \times96485}{8.314 \times303.15}=\text{34.2239}$

$$\frac{p_{H2} \times0.00357}{{({10}^{-7})}^{2}}=e^{34.2239}=7.3 \times{10}^{14}$$

$p_{H2}=\frac{7.3\times{10}^{14} \times{10}^{-14}}{0.00357} =$ $2044 \mathrm{atm}$

Thus, a conservative estimate is that a H_2_ partial pressure greater than 2044 atm (207 Mpa) would be necessary for Fe^0^ oxidation coupled to H_2_ production to be thermodynamically unfavorable.
